# (2*E*,6*E*)-2,6-Bis(2-fluoro-5-meth­oxy­benzyl­idene)cyclo­hexan-1-one

**DOI:** 10.1107/S1600536810048610

**Published:** 2010-11-27

**Authors:** Linfeng Chen, Li Zhang, Zhe Wang, Yunjie Wu, Guang Liang

**Affiliations:** a School of Pharmacy, Wenzhou Medical College, Wenzhou, Zhejiang Province 325035, People’s Republic of China

## Abstract

The title compound, C_22_H_20_F_2_O_3_, a derivative of curcumin, crystallized with two independent mol­ecules in the asymmetric unit. The mean planes of the two 2-fluoro-5-meth­oxy­phenyl groups are aligned at 24.88 (11)° in one mol­ecule and 24.19 (15)° in the other. The dihedral angles between the mean plane of the penta-1,4-dien-3-one group and those of the two 2-fluoro-5-meth­oxy­phenyl rings are 51.16 (11) and 49.16 (10)° in the first mol­ecule, and 45.69 (15) and 54.00 (14)° in the second. The mol­ecules adopt *E* configurations about the central olefinic bonds.

## Related literature

For related structures, see: Liang *et al.* (2007 ▶); Zhao *et al.* (2009 ▶); Zhao, Yang, Liang *et al.* (2010 ▶). For background to and applications of related compounds, see: Aggarwal *et al.* (2003 ▶); Began *et al.* (1999 ▶); Ganesh & Aggarwal (2007) ▶; Liang *et al.*(2009 ▶); Zhao, Yang, Wang *et al.* (2010 ▶).
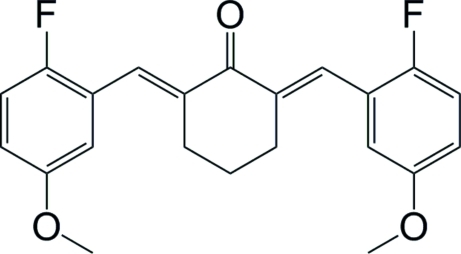


## Experimental

### 

#### Crystal data

C_22_H_20_F_2_O_3_
*M* *_r_* = 370.38
Triclinic, 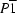

*a* = 9.2334 (10) Å
*b* = 9.7601 (11) Å
*c* = 21.433 (2) Å
α = 90.195 (2)°
β = 100.568 (2)°
γ = 92.934 (2)°
*V* = 1896.1 (4) Å^3^
*Z* = 4
Mo *K*α radiation
μ = 0.10 mm^−1^
*T* = 273 K
0.10 × 0.10 × 0.10 mm

#### Data collection

Bruker APEXII CCD area-detector diffractometer
Absorption correction: multi-scan (*SADABS*; Bruker, 2004 ▶) *T*_min_ = 0.990, *T*_max_ = 0.990
10069 measured reflections
6634 independent reflections
3949 reflections with *I* > 2σ(*I*)
*R*_int_ = 0.101

#### Refinement

*R*[*F* ^2^ > 2σ(*F* ^2^)] = 0.057
*wR*(*F* ^2^) = 0.147
*S* = 1.00
6634 reflections
492 parameters
H-atom parameters not refined
Δρ_max_ = 0.26 e Å^−3^
Δρ_min_ = −0.27 e Å^−3^

### 

Data collection: *APEX2* (Bruker, 2004 ▶); cell refinement: *SAINT* (Bruker, 2004 ▶); data reduction: *SAINT*; program(s) used to solve structure: *SHELXS97* (Sheldrick, 2008 ▶); program(s) used to refine structure: *SHELXL97* (Sheldrick, 2008 ▶); molecular graphics: *SHELXTL* (Sheldrick, 2008 ▶); software used to prepare material for publication: *SHELXTL*.

## Supplementary Material

Crystal structure: contains datablocks I, global. DOI: 10.1107/S1600536810048610/ng5065sup1.cif

Structure factors: contains datablocks I. DOI: 10.1107/S1600536810048610/ng5065Isup2.hkl

Additional supplementary materials:  crystallographic information; 3D view; checkCIF report

## Figures and Tables

**Fig. 1.**
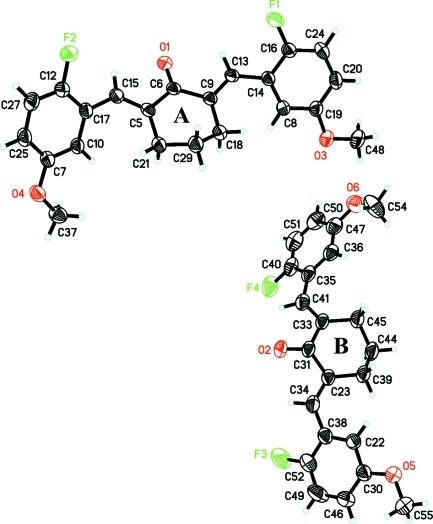
The molecular structure of the title compound, showing 30% displacement ellipsoids for the non-hydrogen atoms. Hydrogen atoms are drawn as spheres of arbitrary radius.

## supplementary crystallographic information

### Comment

The title compound, (2E,6E)-2,6-bis(2-fluoro-5-hydroxybenzylidene)cyclohexanone (I), is one of mono-carbonyl analogues of curcumin designed and synthesized by our group.Curcumin reportedly possesses several pharmacological properties including anti-inflammatory, antimicrobial,antiviral, antifungal, antioxidant, chemosensitizing, radiosensitizing,and wound healing activities.Curcumin can suppress tumor initiation, promotion, and metastasis in experimental models. (Began, *et al.*1999;Ganesh *et al.*2007).Unlike most chemotherapeutic agents, curcumin has been reported to show almost nontoxicity. These compound have attracted more and more attention. (Aggarwal *et al.*2003). The need for curcumin-like compounds with improved bioavailability characteristics has led to the chemical synthesis of a series of analogues, using curcumin as the primary structure. In our previous study, a series of fluorine-containing, mono-carbonyl analogues of curcumin were designed and synthesized by the deletion of β-diketone moiety, and their bioactivities were evaluated (Liang *et al.*, 2009; Zhao *et al.*, 2010). Among those compounds, the cyclohexanone-containing analogues exhibited better anti-tumor properties and a wider anti-tumor spectrum than acetone- and cyclopentanone-containing analogues. As a continuation of our broad program of work on the synthesis and structural study of chalcones, the title chalcone derivative has been obtained and an X-ray diffraction study was carried out. Therefore, the structure of one of cyclohexanone-containing compounds (I), was further determined and analyzed using single-crystal X-ray diffraction. Accumulation of detailed structural and pharmacological data facilitated the explanation of the observed structure–activity relationships and modeling of new compounds with potential biological activity.

In this paper, we report the molecular and crystal structures of fluorine-containing, mono-carbonyl analogues of curcumin, (I). The molecule (I), consists of three ring systems, i.e., one cyclohexanone ring and two aryl rings. The central cyclohexanone ring has a distorted chair conformation, and molecular structures have an *E*-configuration towards the central olefinic bonds, exhibiting a butterfly-shaped geometry. The dihedral angle between the two terminal phenyl rings is 27.19 (13)°, and the two phenyl rings are twisted out of the plane of the central cyclohexanone on the two sides, respectively. Among these derivatives, some of them were reported of their crystal structures ( Liang *et al.*, 2007; Zhao *et al.*, 2009; Zhao *et al.*, 2010).

### Experimental

Cyclohexanone (7.5 mmol) was dissolved in ethanol (5 ml) and crushed KOH (15 mmol) was added. The flask was immersed in a bath of crushed ice and a solution of 2-fluoro-5-hydroxybenzaldehyde (15 mmol) in ethanol (5 mmol) was added. The reaction mixture was stirred at 300 K and completion of the reaction was monitored by thin-layer chromatography. Ice-cold water was added to the reaction mixture after 48 h and the yellow solid that separated was filtered off, washed with water and cold ethanol, dried and purified by column chromatography on silica gel (yield: 58.3%). Single crystals of the title compound were grown in a CH_2_Cl_2_/CH_3_OH mixture (5:2 *v*/*v*) by slow evaporation (mp 91.3-93.4 °C).

Yellow powder, 58.3% yield, mp 91.3-93.4°C. ^1^H-NMR (CDCl_3_) δ: 7.77 (2H, s, Ar-CH=C×2), 7.03 (2H, t, J=9.0Hz, Ar-H^3^×2), 6.83-6.87 (4H, m, Ar-H^4,6^×2), 3.80 (6H, s, Ar-OCH_3_×2), 2.81 (4H, t, J=5.4Hz, CH_2_-C-CH_2_), 1.78 (2H, m, >CH_2_). ESI-MS *m/z*: 371.0 (M+H)^+^, calcd for C_22_H_20_F_2_O_3_: 370.39.

### Refinement

The H atoms were positioned geometrically (C—H = 0.93 and 0.96 Å) and refined as riding with *U*_iso_(H) = 1.2*U*_eq_(C) or 1.5*U*_eq_(methyl C).

### Crystal data

**Table:**

C_22_H_20_F_2_O_3_	*Z* = 4
*M**_r_* = 370.38	*F*(000) = 776
Triclinic, *P*1	*D*_x_ = 1.297 Mg m^−^^3^
Hall symbol: -P 1	Mo *K*α radiation, λ = 0.71073 Å
*a* = 9.2334 (10) Å	Cell parameters from 3025 reflections
*b* = 9.7601 (11) Å	θ = 2.3–23.1°
*c* = 21.433 (2) Å	µ = 0.10 mm^−^^1^
α = 90.195 (2)°	*T* = 273 K
β = 100.568 (2)°	Block, colorless
γ = 92.934 (2)°	0.10 × 0.10 × 0.10 mm
*V* = 1896.1 (4) Å^3^	

### Data collection

**Table:**

Bruker APEXII CCD area-detector diffractometer	6634 independent reflections
Radiation source: fine-focus sealed tube	3949 reflections with *I* > 2σ(*I*)
graphite	*R*_int_ = 0.101
φ and ω scans	θ_max_ = 25.0°, θ_min_ = 1.9°
Absorption correction: multi-scan (*SADABS*; Bruker, 2004)	*h* = −10→10
*T*_min_ = 0.990, *T*_max_ = 0.990	*k* = −11→11
10069 measured reflections	*l* = −18→25

### Refinement

**Table:**

Refinement on *F*^2^	Secondary atom site location: difference Fourier map
Least-squares matrix: full	Hydrogen site location: inferred from neighbouring sites
*R*[*F*^2^ > 2σ(*F*^2^)] = 0.057	H-atom parameters not refined
*wR*(*F*^2^) = 0.147	*w* = 1/[σ^2^(*F*_o_^2^) + (0.055*P*)^2^] where *P* = (*F*_o_^2^ + 2*F*_c_^2^)/3
*S* = 1.00	(Δ/σ)_max_ = 0.015
6634 reflections	Δρ_max_ = 0.26 e Å^−^^3^
492 parameters	Δρ_min_ = −0.27 e Å^−^^3^
0 restraints	Extinction correction: *SHELXL97* (Sheldrick, 2008), Fc^*^=kFc[1+0.001xFc^2^λ^3^/sin(2θ)]^-1/4^
Primary atom site location: structure-invariant direct methods	Extinction coefficient: 0.0102 (12)

### Special details

**Table:**

Geometry. All esds (except the esd in the dihedral angle between two l.s. planes) are estimated using the full covariance matrix. The cell esds are taken into account individually in the estimation of esds in distances, angles and torsion angles; correlations between esds in cell parameters are only used when they are defined by crystal symmetry. An approximate (isotropic) treatment of cell esds is used for estimating esds involving l.s. planes.
Refinement. Refinement of F^2^ against ALL reflections. The weighted R-factor wR and goodness of fit S are based on F^2^, conventional R-factors R are based on F, with F set to zero for negative F^2^. The threshold expression of F^2^ > 2sigma(F^2^) is used only for calculating R-factors(gt) etc. and is not relevant to the choice of reflections for refinement. R-factors based on F^2^ are statistically about twice as large as those based on F, and R- factors based on ALL data will be even larger.

### Fractional atomic coordinates and isotropic or equivalent isotropic displacement parameters (Å^2^)

**Table:**

	*x*	*y*	*z*	*U*_iso_*/*U*_eq_	
F1	0.64586 (15)	−0.51316 (13)	0.03944 (7)	0.0840 (4)	
O1	0.59989 (17)	−0.11281 (16)	−0.08404 (7)	0.0687 (4)	
O4	0.94423 (17)	0.60048 (16)	−0.09902 (8)	0.0769 (5)	
F2	0.94845 (17)	0.08313 (16)	−0.19582 (7)	0.0938 (5)	
C5	0.7533 (2)	0.0813 (2)	−0.04416 (10)	0.0525 (5)	
C6	0.6674 (2)	−0.0492 (2)	−0.03706 (10)	0.0521 (5)	
C7	0.9400 (2)	0.4676 (2)	−0.12065 (11)	0.0602 (6)	
C8	0.5428 (2)	−0.2508 (2)	0.13952 (10)	0.0577 (6)	
H8	0.5357	−0.1562	0.1412	0.069*	
C9	0.6657 (2)	−0.1030 (2)	0.02818 (10)	0.0513 (5)	
C10	0.8601 (2)	0.3605 (2)	−0.09952 (10)	0.0585 (6)	
H10	0.8033	0.3770	−0.0689	0.070*	
O3	0.4590 (2)	−0.25806 (18)	0.23530 (8)	0.0859 (5)	
C12	0.9460 (3)	0.2116 (2)	−0.17015 (11)	0.0666 (6)	
C13	0.6103 (2)	−0.2322 (2)	0.03145 (10)	0.0561 (5)	
H13	0.5820	−0.2785	−0.0072	0.067*	
C14	0.5881 (2)	−0.3100 (2)	0.08760 (10)	0.0541 (5)	
C15	0.7815 (2)	0.1087 (2)	−0.10238 (11)	0.0593 (6)	
H15	0.7441	0.0433	−0.1336	0.071*	
C16	0.6001 (2)	−0.4513 (2)	0.08892 (11)	0.0631 (6)	
C17	0.8626 (2)	0.2266 (2)	−0.12325 (10)	0.0576 (6)	
C18	0.7318 (2)	−0.0142 (2)	0.08455 (10)	0.0603 (6)	
H18A	0.7671	−0.0722	0.1202	0.072*	
H18B	0.6561	0.0409	0.0961	0.072*	
C19	0.5085 (2)	−0.3297 (2)	0.18834 (11)	0.0633 (6)	
C20	0.5209 (3)	−0.4707 (3)	0.18760 (12)	0.0742 (7)	
H20	0.4980	−0.5238	0.2208	0.089*	
C21	0.8076 (3)	0.1678 (2)	0.01397 (10)	0.0664 (6)	
H21A	0.7292	0.2237	0.0222	0.080*	
H21B	0.8891	0.2288	0.0068	0.080*	
C22	0.5255 (3)	0.7126 (3)	0.62792 (12)	0.0742 (7)	
H22	0.5377	0.6186	0.6268	0.089*	
C23	0.3185 (3)	0.5673 (3)	0.51074 (11)	0.0715 (7)	
C24	0.5675 (3)	−0.5308 (3)	0.13702 (13)	0.0727 (7)	
H24	0.5766	−0.6252	0.1358	0.087*	
C25	1.0243 (3)	0.4454 (3)	−0.16668 (12)	0.0723 (7)	
H25	1.0792	0.5179	−0.1805	0.087*	
O5	0.6789 (2)	0.7139 (2)	0.72684 (9)	0.1000 (6)	
C27	1.0266 (3)	0.3162 (3)	−0.19182 (12)	0.0780 (7)	
H27	1.0820	0.3002	−0.2230	0.094*	
O2	0.2913 (2)	0.5903 (2)	0.39992 (8)	0.1026 (6)	
C29	0.8584 (2)	0.0796 (2)	0.07149 (11)	0.0682 (6)	
H29A	0.9384	0.0251	0.0638	0.082*	
H29B	0.8948	0.1382	0.1083	0.082*	
C30	0.5948 (3)	0.7889 (3)	0.68033 (13)	0.0799 (7)	
C31	0.2607 (3)	0.5211 (3)	0.44426 (12)	0.0769 (7)	
F4	−0.1739 (2)	0.4179 (2)	0.28990 (8)	0.1210 (6)	
C33	0.1666 (3)	0.3919 (3)	0.43095 (12)	0.0733 (7)	
C34	0.3691 (3)	0.6978 (3)	0.51894 (12)	0.0803 (7)	
H34	0.3588	0.7492	0.4821	0.096*	
C35	−0.0142 (3)	0.2610 (3)	0.34497 (12)	0.0787 (8)	
C36	0.0179 (3)	0.1235 (3)	0.35708 (12)	0.0826 (8)	
H36	0.1025	0.1030	0.3856	0.099*	
C37	0.8455 (3)	0.6307 (3)	−0.05740 (13)	0.0797 (7)	
H37A	0.7462	0.6047	−0.0775	0.120*	
H37B	0.8534	0.7272	−0.0478	0.120*	
H37C	0.8706	0.5804	−0.0188	0.120*	
C38	0.4382 (3)	0.7727 (3)	0.57692 (13)	0.0776 (7)	
C39	0.3083 (3)	0.4666 (3)	0.56284 (12)	0.0808 (7)	
H39A	0.3055	0.5166	0.6018	0.097*	
H39B	0.3959	0.4139	0.5699	0.097*	
C40	−0.1396 (4)	0.2852 (4)	0.30224 (14)	0.0935 (9)	
C41	0.0852 (3)	0.3759 (3)	0.37287 (13)	0.0840 (8)	
H41	0.0928	0.4493	0.3459	0.101*	
O6	−0.0522 (3)	−0.1187 (2)	0.33634 (11)	0.1141 (7)	
F3	0.3415 (3)	0.97700 (19)	0.53269 (11)	0.1524 (9)	
C44	0.1740 (3)	0.3700 (3)	0.54768 (12)	0.0921 (9)	
H44A	0.0863	0.4217	0.5457	0.111*	
H44B	0.1757	0.3042	0.5816	0.111*	
C45	0.1653 (3)	0.2928 (3)	0.48495 (12)	0.0846 (8)	
H45A	0.2485	0.2349	0.4879	0.102*	
H45B	0.0756	0.2343	0.4766	0.102*	
C46	0.5792 (4)	0.9275 (3)	0.68372 (16)	0.0975 (9)	
H46	0.6264	0.9786	0.7190	0.117*	
C47	−0.0755 (3)	0.0179 (4)	0.32694 (15)	0.0913 (9)	
C48	0.3954 (4)	−0.3343 (3)	0.28049 (13)	0.1087 (10)	
H48A	0.3209	−0.3989	0.2589	0.163*	
H48B	0.3517	−0.2730	0.3060	0.163*	
H48C	0.4704	−0.3826	0.3072	0.163*	
C49	0.4926 (4)	0.9889 (3)	0.6342 (2)	0.1229 (12)	
H49	0.4792	1.0825	0.6358	0.147*	
C50	−0.2011 (4)	0.0478 (4)	0.28457 (16)	0.1095 (11)	
H50	−0.2637	−0.0229	0.2643	0.131*	
C51	−0.2339 (4)	0.1829 (5)	0.27229 (16)	0.1145 (11)	
H51	−0.3189	0.2037	0.2441	0.137*	
C52	0.4259 (4)	0.9131 (3)	0.58215 (17)	0.1020 (9)	
C54	0.0799 (4)	−0.1549 (4)	0.3739 (2)	0.1360 (14)	
H54A	0.1614	−0.1118	0.3581	0.204*	
H54B	0.0861	−0.2527	0.3726	0.204*	
H54C	0.0830	−0.1252	0.4169	0.204*	
C55	0.7647 (4)	0.7839 (3)	0.77809 (15)	0.1175 (11)	
H55A	0.8265	0.8535	0.7630	0.176*	
H55B	0.8252	0.7208	0.8039	0.176*	
H55C	0.7019	0.8258	0.8029	0.176*	

### Atomic displacement parameters (Å^2^)

**Table:**

	*U*^11^	*U*^22^	*U*^33^	*U*^12^	*U*^13^	*U*^23^
F1	0.1075 (10)	0.0526 (8)	0.1018 (11)	0.0057 (7)	0.0447 (9)	−0.0015 (7)
O1	0.0905 (11)	0.0540 (10)	0.0632 (10)	−0.0054 (8)	0.0210 (9)	−0.0035 (8)
O4	0.0902 (11)	0.0533 (10)	0.0936 (12)	−0.0070 (8)	0.0364 (10)	0.0036 (8)
F2	0.1283 (12)	0.0732 (10)	0.0938 (11)	0.0039 (9)	0.0572 (9)	−0.0062 (8)
C5	0.0541 (12)	0.0410 (12)	0.0645 (14)	0.0075 (9)	0.0151 (10)	0.0029 (10)
C6	0.0572 (12)	0.0434 (12)	0.0591 (14)	0.0080 (10)	0.0183 (11)	−0.0030 (10)
C7	0.0629 (13)	0.0544 (15)	0.0648 (14)	0.0012 (11)	0.0163 (11)	0.0092 (11)
C8	0.0638 (13)	0.0511 (13)	0.0594 (14)	−0.0038 (10)	0.0160 (11)	0.0065 (11)
C9	0.0541 (11)	0.0437 (12)	0.0594 (13)	0.0057 (9)	0.0180 (10)	0.0009 (10)
C10	0.0642 (13)	0.0559 (14)	0.0602 (14)	0.0032 (11)	0.0237 (11)	0.0092 (11)
O3	0.1237 (14)	0.0769 (12)	0.0651 (11)	−0.0099 (10)	0.0422 (10)	0.0060 (9)
C12	0.0820 (15)	0.0563 (15)	0.0671 (15)	0.0027 (12)	0.0283 (13)	0.0022 (12)
C13	0.0669 (13)	0.0447 (13)	0.0606 (13)	0.0033 (10)	0.0222 (11)	−0.0028 (10)
C14	0.0573 (12)	0.0472 (13)	0.0603 (14)	0.0004 (10)	0.0178 (10)	0.0058 (10)
C15	0.0664 (13)	0.0504 (13)	0.0651 (15)	0.0049 (10)	0.0222 (11)	0.0045 (11)
C16	0.0705 (14)	0.0533 (14)	0.0684 (15)	0.0007 (11)	0.0213 (12)	0.0024 (12)
C17	0.0617 (13)	0.0541 (14)	0.0598 (14)	0.0024 (10)	0.0189 (11)	0.0093 (11)
C18	0.0670 (13)	0.0535 (13)	0.0616 (14)	0.0004 (11)	0.0158 (11)	0.0040 (11)
C19	0.0717 (14)	0.0625 (16)	0.0565 (14)	−0.0056 (12)	0.0162 (11)	0.0068 (12)
C20	0.0850 (16)	0.0688 (18)	0.0674 (16)	−0.0083 (13)	0.0134 (13)	0.0227 (13)
C21	0.0795 (15)	0.0544 (14)	0.0632 (15)	−0.0096 (11)	0.0108 (12)	0.0057 (11)
C22	0.0987 (18)	0.0635 (16)	0.0681 (17)	0.0113 (14)	0.0334 (14)	0.0018 (13)
C23	0.0922 (17)	0.0703 (18)	0.0614 (16)	0.0245 (14)	0.0337 (13)	0.0114 (12)
C24	0.0839 (16)	0.0494 (14)	0.0856 (18)	0.0006 (12)	0.0185 (14)	0.0153 (13)
C25	0.0761 (15)	0.0691 (17)	0.0772 (17)	−0.0067 (13)	0.0311 (13)	0.0148 (13)
O5	0.1470 (17)	0.0775 (13)	0.0736 (13)	−0.0049 (12)	0.0182 (12)	0.0008 (11)
C27	0.0853 (17)	0.084 (2)	0.0748 (17)	0.0039 (14)	0.0420 (14)	0.0101 (14)
O2	0.1667 (18)	0.0881 (14)	0.0632 (11)	0.0080 (13)	0.0474 (12)	0.0147 (10)
C29	0.0729 (14)	0.0639 (15)	0.0650 (15)	−0.0100 (12)	0.0087 (12)	0.0079 (12)
C30	0.0995 (19)	0.0662 (18)	0.0807 (19)	0.0013 (15)	0.0351 (16)	0.0068 (15)
C31	0.1073 (19)	0.0776 (18)	0.0566 (15)	0.0314 (15)	0.0355 (14)	0.0123 (13)
F4	0.1282 (13)	0.1394 (17)	0.1007 (12)	0.0539 (12)	0.0215 (10)	0.0341 (11)
C33	0.0932 (17)	0.0785 (19)	0.0570 (16)	0.0277 (15)	0.0305 (14)	0.0111 (13)
C34	0.1038 (19)	0.080 (2)	0.0666 (17)	0.0287 (16)	0.0325 (14)	0.0184 (14)
C35	0.0879 (18)	0.098 (2)	0.0588 (15)	0.0259 (17)	0.0311 (14)	0.0082 (15)
C36	0.0878 (18)	0.099 (2)	0.0680 (17)	0.0251 (17)	0.0271 (14)	0.0042 (16)
C37	0.0767 (15)	0.0667 (17)	0.101 (2)	0.0059 (13)	0.0291 (15)	−0.0043 (14)
C38	0.0963 (18)	0.0647 (17)	0.0798 (19)	0.0160 (14)	0.0339 (15)	0.0086 (14)
C39	0.0998 (18)	0.089 (2)	0.0597 (15)	0.0095 (16)	0.0302 (14)	0.0125 (13)
C40	0.097 (2)	0.122 (3)	0.0701 (19)	0.041 (2)	0.0291 (17)	0.0216 (19)
C41	0.107 (2)	0.088 (2)	0.0671 (18)	0.0394 (17)	0.0333 (16)	0.0119 (15)
O6	0.1315 (18)	0.0947 (18)	0.1233 (18)	0.0054 (14)	0.0425 (15)	−0.0041 (13)
F3	0.196 (2)	0.0803 (13)	0.1656 (19)	0.0409 (13)	−0.0178 (16)	0.0237 (12)
C44	0.113 (2)	0.104 (2)	0.0666 (17)	0.0101 (18)	0.0360 (15)	0.0221 (15)
C45	0.1054 (19)	0.088 (2)	0.0659 (17)	0.0096 (15)	0.0288 (14)	0.0173 (14)
C46	0.123 (2)	0.071 (2)	0.099 (2)	−0.0025 (18)	0.0245 (19)	−0.0082 (17)
C47	0.091 (2)	0.110 (3)	0.081 (2)	0.011 (2)	0.0364 (17)	−0.0005 (19)
C48	0.162 (3)	0.104 (2)	0.0699 (18)	−0.014 (2)	0.0522 (19)	0.0138 (16)
C49	0.146 (3)	0.058 (2)	0.162 (4)	0.013 (2)	0.020 (3)	−0.005 (2)
C50	0.101 (2)	0.137 (3)	0.093 (2)	0.001 (2)	0.026 (2)	−0.006 (2)
C51	0.098 (2)	0.158 (4)	0.088 (2)	0.019 (3)	0.0149 (19)	0.012 (2)
C52	0.125 (2)	0.070 (2)	0.111 (2)	0.0194 (19)	0.016 (2)	0.0157 (19)
C54	0.109 (3)	0.091 (3)	0.218 (4)	0.025 (2)	0.049 (3)	0.038 (3)
C55	0.166 (3)	0.104 (3)	0.076 (2)	−0.010 (2)	0.009 (2)	−0.0054 (18)

### Geometric parameters (Å, °)

**Table:**

F1—C16	1.362 (3)	O2—C31	1.234 (3)
O1—C6	1.231 (2)	C29—H29A	0.9700
O4—C7	1.372 (3)	C29—H29B	0.9700
O4—C37	1.427 (3)	C30—C46	1.371 (4)
F2—C12	1.371 (3)	C31—C33	1.490 (4)
C5—C15	1.346 (3)	F4—C40	1.364 (4)
C5—C6	1.489 (3)	C33—C41	1.335 (3)
C5—C21	1.495 (3)	C33—C45	1.512 (3)
C6—C9	1.498 (3)	C34—C38	1.464 (4)
C7—C10	1.375 (3)	C34—H34	0.9300
C7—C25	1.387 (3)	C35—C40	1.370 (4)
C8—C19	1.375 (3)	C35—C36	1.404 (4)
C8—C14	1.392 (3)	C35—C41	1.465 (4)
C8—H8	0.9300	C36—C47	1.387 (4)
C9—C13	1.343 (3)	C36—H36	0.9300
C9—C18	1.501 (3)	C37—H37A	0.9600
C10—C17	1.404 (3)	C37—H37B	0.9600
C10—H10	0.9300	C37—H37C	0.9600
O3—C19	1.381 (3)	C38—C52	1.386 (4)
O3—C48	1.416 (3)	C39—C44	1.504 (4)
C12—C27	1.366 (3)	C39—H39A	0.9700
C12—C17	1.385 (3)	C39—H39B	0.9700
C13—C14	1.465 (3)	C40—C51	1.368 (5)
C13—H13	0.9300	C41—H41	0.9300
C14—C16	1.389 (3)	O6—C47	1.370 (4)
C15—C17	1.457 (3)	O6—C54	1.395 (4)
C15—H15	0.9300	F3—C52	1.368 (3)
C16—C24	1.361 (3)	C44—C45	1.526 (4)
C18—C29	1.515 (3)	C44—H44A	0.9700
C18—H18A	0.9700	C44—H44B	0.9700
C18—H18B	0.9700	C45—H45A	0.9700
C19—C20	1.387 (3)	C45—H45B	0.9700
C20—C24	1.378 (3)	C46—C49	1.367 (4)
C20—H20	0.9300	C46—H46	0.9300
C21—C29	1.522 (3)	C47—C50	1.381 (4)
C21—H21A	0.9700	C48—H48A	0.9600
C21—H21B	0.9700	C48—H48B	0.9600
C22—C30	1.381 (3)	C48—H48C	0.9600
C22—C38	1.385 (3)	C49—C52	1.366 (5)
C22—H22	0.9300	C49—H49	0.9300
C23—C34	1.334 (4)	C50—C51	1.383 (5)
C23—C31	1.486 (4)	C50—H50	0.9300
C23—C39	1.502 (3)	C51—H51	0.9300
C24—H24	0.9300	C54—H54A	0.9600
C25—C27	1.372 (3)	C54—H54B	0.9600
C25—H25	0.9300	C54—H54C	0.9600
O5—C30	1.383 (3)	C55—H55A	0.9600
O5—C55	1.386 (3)	C55—H55B	0.9600
C27—H27	0.9300	C55—H55C	0.9600
			
C7—O4—C37	116.94 (17)	C23—C31—C33	120.4 (2)
C15—C5—C6	116.80 (19)	C41—C33—C31	117.1 (2)
C15—C5—C21	125.17 (19)	C41—C33—C45	125.2 (3)
C6—C5—C21	117.96 (19)	C31—C33—C45	117.6 (2)
O1—C6—C5	120.62 (19)	C23—C34—C38	130.1 (2)
O1—C6—C9	120.33 (18)	C23—C34—H34	115.0
C5—C6—C9	119.04 (19)	C38—C34—H34	115.0
O4—C7—C10	124.4 (2)	C40—C35—C36	117.3 (3)
O4—C7—C25	115.3 (2)	C40—C35—C41	120.1 (3)
C10—C7—C25	120.3 (2)	C36—C35—C41	122.6 (3)
C19—C8—C14	121.1 (2)	C47—C36—C35	120.6 (3)
C19—C8—H8	119.4	C47—C36—H36	119.7
C14—C8—H8	119.4	C35—C36—H36	119.7
C13—C9—C6	116.31 (19)	O4—C37—H37A	109.5
C13—C9—C18	124.71 (19)	O4—C37—H37B	109.5
C6—C9—C18	118.90 (18)	H37A—C37—H37B	109.5
C7—C10—C17	121.2 (2)	O4—C37—H37C	109.5
C7—C10—H10	119.4	H37A—C37—H37C	109.5
C17—C10—H10	119.4	H37B—C37—H37C	109.5
C19—O3—C48	118.0 (2)	C52—C38—C22	115.7 (3)
C27—C12—F2	118.2 (2)	C52—C38—C34	120.7 (3)
C27—C12—C17	124.2 (2)	C22—C38—C34	123.5 (2)
F2—C12—C17	117.6 (2)	C23—C39—C44	112.5 (2)
C9—C13—C14	128.9 (2)	C23—C39—H39A	109.1
C9—C13—H13	115.6	C44—C39—H39A	109.1
C14—C13—H13	115.6	C23—C39—H39B	109.1
C16—C14—C8	116.4 (2)	C44—C39—H39B	109.1
C16—C14—C13	120.4 (2)	H39A—C39—H39B	107.8
C8—C14—C13	122.94 (19)	F4—C40—C51	118.3 (3)
C5—C15—C17	129.0 (2)	F4—C40—C35	118.5 (3)
C5—C15—H15	115.5	C51—C40—C35	123.2 (3)
C17—C15—H15	115.5	C33—C41—C35	130.2 (3)
C24—C16—F1	118.5 (2)	C33—C41—H41	114.9
C24—C16—C14	123.2 (2)	C35—C41—H41	114.9
F1—C16—C14	118.3 (2)	C47—O6—C54	118.4 (3)
C12—C17—C10	115.72 (19)	C39—C44—C45	112.5 (2)
C12—C17—C15	120.5 (2)	C39—C44—H44A	109.1
C10—C17—C15	123.8 (2)	C45—C44—H44A	109.1
C9—C18—C29	112.16 (18)	C39—C44—H44B	109.1
C9—C18—H18A	109.2	C45—C44—H44B	109.1
C29—C18—H18A	109.2	H44A—C44—H44B	107.8
C9—C18—H18B	109.2	C33—C45—C44	110.8 (2)
C29—C18—H18B	109.2	C33—C45—H45A	109.5
H18A—C18—H18B	107.9	C44—C45—H45A	109.5
C8—C19—O3	114.9 (2)	C33—C45—H45B	109.5
C8—C19—C20	120.6 (2)	C44—C45—H45B	109.5
O3—C19—C20	124.4 (2)	H45A—C45—H45B	108.1
C24—C20—C19	119.0 (2)	C49—C46—C30	118.6 (3)
C24—C20—H20	120.5	C49—C46—H46	120.7
C19—C20—H20	120.5	C30—C46—H46	120.7
C5—C21—C29	111.31 (19)	O6—C47—C50	116.0 (3)
C5—C21—H21A	109.4	O6—C47—C36	124.1 (3)
C29—C21—H21A	109.4	C50—C47—C36	119.9 (3)
C5—C21—H21B	109.4	O3—C48—H48A	109.5
C29—C21—H21B	109.4	O3—C48—H48B	109.5
H21A—C21—H21B	108.0	H48A—C48—H48B	109.5
C30—C22—C38	121.5 (3)	O3—C48—H48C	109.5
C30—C22—H22	119.3	H48A—C48—H48C	109.5
C38—C22—H22	119.3	H48B—C48—H48C	109.5
C34—C23—C31	116.4 (2)	C46—C49—C52	120.1 (3)
C34—C23—C39	125.6 (2)	C46—C49—H49	119.9
C31—C23—C39	117.9 (2)	C52—C49—H49	119.9
C16—C24—C20	119.6 (2)	C47—C50—C51	120.1 (4)
C16—C24—H24	120.2	C47—C50—H50	120.0
C20—C24—H24	120.2	C51—C50—H50	120.0
C27—C25—C7	119.9 (2)	C40—C51—C50	119.0 (3)
C27—C25—H25	120.1	C40—C51—H51	120.5
C7—C25—H25	120.1	C50—C51—H51	120.5
C30—O5—C55	118.4 (2)	C49—C52—F3	119.2 (3)
C12—C27—C25	118.7 (2)	C49—C52—C38	123.1 (3)
C12—C27—H27	120.7	F3—C52—C38	117.7 (3)
C25—C27—H27	120.7	O6—C54—H54A	109.5
C18—C29—C21	110.31 (18)	O6—C54—H54B	109.5
C18—C29—H29A	109.6	H54A—C54—H54B	109.5
C21—C29—H29A	109.6	O6—C54—H54C	109.5
C18—C29—H29B	109.6	H54A—C54—H54C	109.5
C21—C29—H29B	109.6	H54B—C54—H54C	109.5
H29A—C29—H29B	108.1	O5—C55—H55A	109.5
C46—C30—O5	124.6 (3)	O5—C55—H55B	109.5
C46—C30—C22	121.0 (3)	H55A—C55—H55B	109.5
O5—C30—C22	114.4 (2)	O5—C55—H55C	109.5
O2—C31—C23	119.6 (3)	H55A—C55—H55C	109.5
O2—C31—C33	119.9 (2)	H55B—C55—H55C	109.5
